# Where do we Stand in the Historiography of Small Disciplines in Nazi Germany? The Case of Indology

**DOI:** 10.1007/s00048-023-00365-y

**Published:** 2023-09-11

**Authors:** Moritz Epple, Maria Framke, Eli Franco, Horst Junginger, Baijayanti Roy

**Affiliations:** 1 Goethe-Universität, Historisches Seminar, Norbert-Wollheim-Platz 1, 60629 Frankfurt am Main, Germany; 2 Universität Erfurt, Historisches Seminar, Nordhäuser Str. 63, 99089 Erfurt, Germany; 3 Universität Leipzig, Institut für Indologie und Zentralasienwissenschaften, Schillerstraße 6, 04109 Leipzig, Germany; 4 Universität Leipzig, Religionswissenschaftliches Institut, Schillerstraße 6, 04109 Leipzig, Germany

From the moment the NSDAP entered the German government and the first new regulations began to affect academics, critical comments on the role and involvement of Germany’s professors and scientists in the new regime began to appear.^1^ Not surprisingly, these critical voices were countered, and often drowned out, by apologetic statements (in speech or writing) from the very group criticized. After the defeat of Nazi Germany, both these critiques and their apologetic replies were gradually enriched by historical studies of very different kinds. An initial, and indeed rather mixed, type of historical account emerged from the Allied investigation teams, who travelled through Germany to interview scientists about their war efforts and invited some of them to write their own reports about the research done during the war.^2^ This body of texts still remains one of the major sources for historical research on science and medicine in Nazi Germany. Moreover, scholars driven out of Germany told their stories in autobiographies and interviews; some historians have then taken up the leads offered by these accounts.^3^ Simultaneously, and in contrast to this early historical literature on science in Nazi Germany, the autobiographical genre also provided a space for many German academics whose careers had profited from the Third Reich to ‘purify’ and depoliticize their own trajectories.^4^

It has often been discussed that it took several decades before this very uneven historical writing was supplemented by historical studies that reached a level of scholarly precision and critical depth. In Germany, serious historical research and debate had to wait until both the generation of academics who were active during the Nazi period and the generation of their loyal students had retired from positions of academic influence. However, in the last four decades, substantial scholarship has emerged for most major academic fields from the sciences to the humanities. Today, this scholarship provides an understanding of the main lines and patterns of both the persecution of academics in Nazi Germany and the involvement of those who remained in the country, in German social life and the institutions of the German state from 1933 to 1945.

However, with regard to small disciplines, be it in the humanities or in the sciences, the situation remains much more uneven. While some small fields have received careful critical study,^5^ others remain stuck in the divide between isolated critical accounts and apologetic attempts to avoid historical scrutiny. Several issues contribute to this situation: For one, it is understandable that historians, themselves often not trained in the disciplines under study, have focused their efforts on larger fields whose role for academic life in Nazi Germany is more obvious. Moreover, within small disciplines, only a very limited number of younger scholars have turned a critical eye on their own field in Nazi Germany, since in smaller fields more depended and depends on a few select individuals and direct academic lineages. For this reason, members of small fields approaching this history in a critical way may still fear for the progress of their careers. In addition, the subject matter of the fields themselves can throw up very specific obstacles.

In order to shed light on these problems, we first outline a general pattern of the development of historical research on the Nazi past of an academic discipline, before turning to the obstacles specific to Indology, which nonetheless exemplify problems that can (but do not always) occur for small fields in the humanities.

## Four Phases of Research in Germany on the Nazi Past of an Academic Field

Looking at the history of other academic disciplines and institutions under Nazi rule, as it has been conducted in recent decades within Germany, one can discern a pattern of (at least) four distinct phases of critical engagement with the past, in terms of both the nature of the research and the role of various groups participating in and reacting to that research.^6^

As indicated above, the *first phase* has often been prefaced by isolated biographical and at times autobiographical work, often by members of the discipline or institutions concerned. Some of this work recalled the careers of persecuted and dismissed scholars, while other texts contributed to selectively smoothing or ‘purifying’ the biographies of scholars whose careers remained unhindered if not helped by the German state during the years of Nazi rule. Against this backdrop, the first phase of critical research was often triggered by the aspirations of a few isolated researchers (sometimes individual members of the discipline, sometimes a new generation of historians of the field, often at an early stage in their career). More often than not, this new generation was met (in Germany) with a concerted effort by a majority of members of the discipline in question to block their critical scholarship. To give just one example: When one of the authors of this forum began to engage with the history of mathematics in Nazi Germany, he was confronted with one instance of the inner workings of this concerted obstruction. In a still unexplored archival holding, he found a letter, written in the early 1980s, from an older member of the discipline to the director of an institution that (illegitimately) held a large body of relevant archival materials. The letter suggested that even the existence of such materials should be concealed from a particular historian of mathematics who had hoped to take a closer look at the history of the German Mathematical Society and its president Wilhelm Süss between 1937 and 1945. When, in the mid-1990s, a closer examination of these materials finally became possible, it became clear that significant portions of the materials had been removed from the holdings over the years. In talking to this first generation of critical historians about their interactions with older members of the discipline, we also learned that threats about possible damage to one’s career were not uncommon.^7^

The *second phase* is then marked by the gradual emergence of a larger number of critical studies of individual episodes and careers in the discipline, field or institution under consideration by various historians, including members of the discipline. During this phase, two tendencies often compete within the discipline in question: while a tendency still exists to resist critical enquiry among some members of the discipline, other members develop a certain degree of acceptance of critical research. This phase is often characterized by the emergence of very valuable scholarship, and while it is no longer possible to block this research entirely (as attempted in the first phase), there is still no comprehensive understanding of the workings of the field of knowledge as a whole.

The *third phase*, we would say, provides exactly that—detailed and broad archival research by numerous historians and supported by influential members of the discipline, leading to a coherent and nuanced historical scholarship that can no longer be contested. In all the cases we are aware of, this phase not only produces detailed narratives on the basis of a comprehensive analysis of archival evidence and the relevant historiographical context, but also provides new categories of analysis and an awareness of the structural features specific to the field in question. This phase is expensive, and thus dependent both on institutional and financial support. Researchers require funding for several years to do this kind of work, and archival materials must be fully accessible at all relevant institutions. One the most significant examples of work completed in this phase is the impressive collaborative research project on the history of the Kaiser Wilhelm Society, funded by the Max Planck Society over a number of years. The many books and studies that have emerged from this work are a reference for all of us doing similar work in other areas.^8^

For individual disciplines or institutions, the third phase includes, at a minimum, a full survey of the persecution of scholars perceived as undesirable and its effects on the intellectual and social dynamics of the discipline or institution. This phase should also produce a full and a comprehensive analysis of the forms and functions of what Herbert Mehrtens has termed the “relations of collaboration” between individual scholars and the German state, from ideological support for Nazi doctrines to working with and for the government or Nazi party organizations, and actual involvement in Nazi crimes.

In principle, one would wish that this phase be followed by discussions about how to adjust both the memories of persecution and the policies of research and disciplinary discourse in the fields concerned, with the aim of preventing a similar dynamic in the future. Nonetheless, it must be noted that after these three phases, which cumulatively lead to substantial progress in the production of historical insight, there sometimes comes a *fourth phase*, consisting of a new round of debates aimed at relativizing the results of the third phase, both among members of the discipline under consideration and among historians. These new debates can be quite complex, and may include the renewed potential for a backlash against critical research. The number of new historical studies decreases, and it will be interesting to see the future of such debates.

With regard to this pattern of consecutive phases of both the debates and the research surrounding different academic disciplines in Nazi Germany, it is clear that the situation is neither even nor equal. For certain disciplines, including medicine and physics, as well as major institutions such as the Kaiser Wilhelm Society or the Deutsche Forschungsgemeinschaft, we are currently in phase four. Others, including the life sciences, mathematics and certain disciplines in the humanities, scholars are still working towards the completion of the third phase. In quite a few other fields, we are still stuck in phase two, or even in phase one.

## Obstacles

Where do we stand in terms of the history of Indology, and of South Asian Studies, during the Nazi period? Based on the state of research provided in our introduction, it is clear that Sheldon Pollock’s seminal article—and the reactions to it—constitute a typical example of what happens in phase one. Thanks to the work of many scholars, phase two has been in progress for quite some time, although we cannot claim to have made substantial progress towards phase three yet.

In contrast to the best contributions to this second phase, it is important to note the silence, if not resistance, of many representatives of the academic discipline of Indology to Pollock’s challenge. In German Indological circles, Pollock’s essay and a related call by the Indologist Jan E.M. Houben to look critically at Indology’s past during a conference of Orientalists in Leipzig in 1995,^9^ have received considerable critique, as well as outright rejection. Among Indologists, Reinhold Grünendahl has repeatedly claimed that Pollock misconstrued the nature of German Indology, misinterpreted the evidence and failed to recognize that German Indology was an objective *Wissenschaft *impervious to ideological influences (Grünendahl 2006 and 2012).^10^ In 2008, Jürgen Elvert and Jürgen Nielsen-Sikora, two historians at the University of Cologne, published a volume on *Kulturwissenschaften im Nationalsozialismus *(Cultural Studies in National Socialism). This collective enterprise included an impressive twenty-eight university disciplines (including religious studies, see Junginger 2009). Indology, however, did not figure among them. The editors disclosed to one of the authors that their vigorous efforts to find an Indologist willing to contribute were met only with reluctance or disapproval—whichever door they knocked on hoping for co-operation.^11^ Very few professors of Indology working in Germany have been willing to take a closer look at the Nazi past of their discipline, at least in their own departments. Of the few, Heidrun Brückner, mentioned in the introduction, organized a workshop at the University of Würzburg in 2008 that met with little response; the lectures on Indology in Nazi Germany given at this workshop failed to be published.^12^

Quite obviously, an older strategy based on apologetics still retains some power over the field. During the 1950s and 1960s, many scholars in the humanities reacted to their previous ideological involvement in Nazi Germany by taking refuge in the image of an ostensibly apolitical occupation analysing untainted texts in and for themselves. Similarly for the natural sciences, large segments of the academic system in West Germany had long pushed the notion of an “apolitical” science (in a move that was obviously political in itself).^13^ Yet, while most German university disciplines began to move away from their previous unwillingness to engage with their past in the 1970s, Indology continued to cloister itself from the increasing and ultimately decisive success of research on the Nazi past in other areas of the history of the sciences and humanities. Many of Indology’s scholars once again claimed an “irresponsible purity”—to use Herbert Mehrtens’s fitting term—for its (often brilliant) textual studies and denied any ideological commitments in tune with and in the service of the German state between 1933 and 1945.

In January 2007, a rather bizarre discussion began on the international Indology mailing list hosted by the University of Liverpool, under the heading “Indology and “the disastrous ideology of the ‘pure Aryan race’”.^14^ As it turned out, many of the contributors held surprising views of how historical scholarship works. Some considered arguments about the discipline’s Nazi past that were based on previous historical investigation to be an assault on the respectability of the discipline. Critical scholarship that had emerged since Pollock’s article was accused of nest-fouling, and young researchers were denounced as envious faultfinders who were only trying to censure their teachers. The opposition to critical historical research even led some participants of the debate into the dead end of revisionist history.^15^

No historian today will claim that an understanding of the issues discussed here is possible without studying the archives. While personal and institutional files were largely inaccessible until the 1980s, and certain access restrictions remain in place even today, one can no longer base judgments about the political role of Indology in Nazi Germany on a selective reading of contributions to philological studies of Sanskrit texts alone. The ongoing refusal to connect the play on the stage, so to speak, with the scenery behind it, has led Indology to take up an outsider position in academe.

A further complication of these ongoing debates is very specific to the field of Indology. It arises from a rejection of Sheldon Pollock’s second claim, namely that the affinity of German Indology during the Third Reich to German politics might have been related to pre-colonial motifs of Aryan supremacy in the Sanskrit tradition. Not surprisingly, this claim was attacked by present-day defenders of Hindu nationalism. Among those who initially defended Pollock’s critical view of German Indology during the Nazi period, mention must be made of Vishwa Adluri, whose article in support of some of Pollock’s claims (Adluri 2011) was also disparaged by Grünendahl in 2012.^16^ However, a new round of scholarly controversy was sparked by Adluri and Joydeep Bagchee’s 2014 book, *The Nay Science: A History of German Indology*. Firmly rejecting Pollock’s thoughts on elements of pre-colonial domination in the Sanskrit tradition, the book claimed, based on examples of the reception history of the *Mahābhārata* and *Bhagavad Gītā* by generations of German scholars, that the German scholarship on India had long been characterized by incipient racism and colonialist attitudes. The authors thus claimed that any affinity with Nazi politics could in no way be blamed on Sanskrit tradition itself. The book prompted substantial critique from various Indologists, including Eli Franco and Jürgen Hanneder (see Franco 2016; Hanneder 2017). Once again, Reinhold Grünendahl’s response, entitled *Pseudodoxia postorientalis: Erkundungen eines amerikanischen Diskurses über die Indienrezeption in der Wilhelminischen Kaiserzeit (1871–1918)*, published in 2019, can be seen as a comprehensive attempt to stem all discussion on the more problematic legacies of Indian studies in Germany.^17^ While it is certainly legitimate to raise the question of colonialist and racist elements in the tradition of German Indology,^18^ this should not detract from a focus on *both* issues raised in Pollock’s 1993 contribution. In the present special issue, we will however stay clear of this second array of issues and restrict our attention to academic expertise and popular knowledge about India in Germany in the period 1933 to 1945. It is our hope that the pages of this issue generate an awareness that progress into the third phase of research into the history of Indology and Indian studies in Nazi Germany is both needed and possible.

Returning to the larger question of small disciplines, what can be learned from the case of Indology? In addition to the contingencies caused by the small number of interested historians and members of any such field, similar obstacles to the ones described here likely still exist for other small fields, especially in the humanities. This is true in particular whenever ideological motives prevalent in such disciplines were offered to and picked up by political and public discourses during the Nazi period, such as those tied to ‘Aryanism’. After all, one of the functions of most if not all disciplines in the humanities, and not only in Nazi Germany, may be to provide useful ‘ideologemes’ to the governments funding their research.

## Footnotes

1 A telling example of such early accounts is the collection put together in exile by Emil Julius Gumbel (1938). Gumbel’s introduction to that volume, entitled “Die Gleichschaltung der deutschen Hochschulen”, is still worth reading.

2 The main, but by no means only, published collection of such investigation reports is the FIAT Review of German Science and Medicine (Wiesbaden: W. Klemm [for] Office of Military Govt. for Germany, Field Information Agencies Technical, British, French, U.S., 1947–1948). Many other similar reports remain unpublished and can be traced in various archives.

3 For one of the earliest such historical accounts, see Weinreich (1946).

4 For a characteristic and telling example, see the strongly romanticizing autobiography of aerodynamicist Walter Georgii, *Forschen und Fliegen: Ein Lebensbericht*, Tübingen: Schlichtenmayer, 1954. Georgii was one of the four members of the so-called *Forschungsführung* in the Reich Aviation Ministry, overseeing all war-related research funded by the ministry from 1942 until the end of the war.

5 To mention but one example: Fabian Link’s exemplary study of *Burgenforschung* (2014), at the intersection of general history and archaeology.

6 The following is based on our understanding of the historiography not only of the history of major disciplines in the humanities, such as history, and the social sciences in this period, but also of various fields in the natural sciences and engineering, including (but not restricted to) physics, mathematics and mathematical engineering, in particular aviation research, the history of the life sciences and medicine, and collective research projects, such as on the history of the Kaiser Wilhelm Society during the Nazi period and the history of the Deutsche Forschungsgemeinschaft, as well as the institutional history of universities in this period. Over the years, various members of our Frankfurt group have contributed research to several of these areas; presently, a history of applied entomology in Nazi Germany is the subject of a new and ongoing research project. It must be added that *outside* Germany, the development of research on the history of the sciences and the humanities in Nazi Germany has, understandably, followed a different route with different phases, and encountering a different set of obstacles.

7 The institute involved here is the Mathematisches Forschungsinstitut Oberwolfach; the letter is from Emmanuel Sperner to Martin Barner, then director of the MFO, about the historian Herbert Mehrtens. See Kneser/Epple/Speck (1997) and Epple (2006). Since then, the archival materials in question have been transferred to the University Archive Freiburg and are accessible to researchers; however, the content of the letter remains restricted and cannot be quoted here. Michael Kater’s experiences are another telling example, see the introduction to this special issue, note 7.

8 The proceedings (Kaufmann 2000) of the opening conference of this enterprise provide a very valuable survey of the state of research on the history of science in National Socialist Germany at that time. Rather than listing a selection of the titles that resulted from this research program, we refer interested readers to the informative essay review of the entire series (Ash 2010).

9 See Houben’s interesting report (1996). See also the details in Houben’s later Postscripts to this report, available at https://www.academia.edu/7378413 (last accessed 11 February 2022).

10 Grünendahl, now retired, worked as a librarian and researcher at the Niedersächsische Staats- und Universitätsbibliothek Göttingen. The attempt to dissociate Indology as pure *Wissenschaft* from political servility also informs Jürgen Hanneder’s apologetic account of Indology in Marburg during National Socialism (Hanneder 2010), see in particular on pages 47–48. That Hanneder sides with Grünendahl against Pollock (and Edward Said) is equally clear (Hanneder 2013). Walter Slaje’s defence of Erich Frauwallner (Slaje 2010), discussed in Eli Franco’s contribution to this special issue, argues along similar lines.

11 Personal communication with Horst Junginger.

12 Horst Junginger’s contribution to the Würzburg workshop, entitled “Terra incognita: Die Geschichte der Indologie in der Zeit des Nationalsozialismus”, is available in manuscript form from the author. A second contribution to the workshop was a talk by Frank Neubert on the controversy between Hertel and Ludendorff. A revised form was eventually published (Neubert 2012).

13 For the mathematical sciences, see Mehrtens’s exemplary study, wherein he analyzed this strategy as one of “irresponsible purity” (1994). For Werner Heisenberg, often considered to be one of the major exponents of “apolitical” science, see Carson (1999). Much earlier, the point was also made for West German academia as a whole by Wolfgang Abendroth (1966).

14 This list, https://list.indology.info/mailman/listinfo/indology, possesses a searchable archive, see https://list.indology.info/pipermail/indology/. The discussion can be traced in this archive from January 2007 onwards. When, in May 2007, Jan E.M. Houben began a separate thread entitled “Essential Reading on Nazi-time European Indology”, he started it with stating “A. Full fledged studies of Nazi-time European Indology—nil”. See https://list.indology.info/pipermail/indology/2007-May/030742.html (last accessed 11 February 2022).

15 While discussions on mailing lists are transient communications and cannot easily be compared with scholarly publications, they do sometimes provide—as in this case—a telling window onto the state and development of disciplinary discourses.

16 In this article (Grünendahl 2012), the author also opposed Houben’s call to look critically at Indology’s past during the conference of Orientalists at Leipzig in 1995, see note 9 above.

17 Grünendahl’s online post on 15 December 2020 (on the German list Indologie@listserv.uni-heidelberg.de) criticizing Eli Franco’s recently published article on the Schlegel brothers and their interest in India is another indication that controversies regarding the definition of Indology and aspects of its past are far from over. For an extensive response to Grünendahl, see Franco (2021).

18 Sheldon Pollock would certainly agree. For nineteenth-century developments in German Orientalism, see the literature cited in note 1 of the introduction to this special issue.
